# Supplementary material for: Multi-Omics Approach Profiling Metabolic Remodeling in Early Systolic Dysfunction and in Overt Systolic Heart Failure
Source: Int J Mol Sci. 2021 Dec 26;23(1):235. doi: 10.3390/ijms23010235 (PMC8745344; doi:10.3390/ijms23010235)
Supplement: Supplementary file 1 [file ijms-23-00235-s001.zip › ijms-23-00235-s001/ijms-1476098 SUPPLEMENTAL MATERIAL.pdf]

SUPPLEMENTAL MATERIAL

Discovery-based proteomic and p-proteomic analysis using tandem mass tags and liquid chromatography mass spectrometry

Protein Extraction. Frozen tissue pieces were ground on dry ice using a CryoGrinder™ (OPS Diagnostics, Lebanon, NJ). Tissue samples were weighed in separate 1.5 mL Eppendorf LoBind tubes and 10ul/mg of extraction buffer was added [7 M urea, 2 M thiourea, 0.4 Tris pH 8, 20% acetonitrile, 4 mM tris (2-carboxyethyl) phosphine (TCEP), and 1X HALT protease & phosphatase inhibitor (ThermoFisher Scientific)]. The samples were sonicated at 30% amplitude for 7 seconds with a Branson Digital Sonifier 250 (Branson Ultrasonics, Danbury, CT). Each sample was transferred to a PCT tube for the Barocycler NEP2320 (Pressure Biosciences, Inc., South Easton, MA) and cycled between 35 kPSI for 20 sec. and 0 kPSI for 10 sec. for 60 cycles at 37 °C. After barocycling, 200 mM chloroacetamide was added to a final concentration of 8 mM chloroacetamide and incubated 15 minutes at room temperature. Samples were centrifuged at 13000g for 10 min. The samples were transferred to a new 1.5 mL microfuge Eppendorf Protein LoBind tube. Aliquots for each sample were taken for protein concentration determination by Bradford assay.

In-Solution Proteolytic Digestion and TMT10plex™ Isobaric Labeling. A 210 µg aliquot of each sample was transferred to a new 1.5 mL microfuge tube and brought to the same volume with protein extraction buffer plus 8 mM chloroacetamide. All samples were diluted fivefold with water, and then trypsin (Promega, Madison, WI) was added in a 1:40 ratio of trypsin to total protein. Samples were incubated overnight for 16 hrs at 37 °C. After incubation, each sample was acidified to 0.3% TFA. Each sample was cleaned with a 1 mL Waters Oasis HLB (Waters Corporation, Milford, MA), eluates were vacuum dried and resuspended in 100µl 0.1 M triethylammonium bicarbonate, pH 8.5, to a final concentration of 2 µg/µl. For each channel within a TMT10plex™ a 200 µg aliquot of each sample was made, and the samples were labeled with the corresponding TMT10plex™ Isobaric Label Reagent, 0.8 mg (Thermo Scientific, Watham, MA) per the manufacturer's protocol. After labeling, all the samples within the same TMT™ experiment were multiplexed together into a new 1.5 mL microfuge tube. To look at the global proteome and phosphorylated proteome, the pooled TMT™ sample was split 10:90 global:phospho, respectively, to provide an aliquot for global TMT™ protein quantification and an aliquot for phosphopeptide TMT™ quantification.

Global TMT™ Sample Fractionation. The aliquoted TMT™ sample for global protein analysis was dried down *in vacuo*. The sample was cleaned with a 1 mL Oasis C18 solid phase extraction cartridge (Waters Corporation, Milford, MA) and the eluate was dried *in vacuo*. The sample was resuspended in Buffer A (20 mM ammonium formate, pH 10, in 98:2 water:acetonitrile) and fractionated offline by high pH C18 reversed-phase (RP) chromatography[95]. High-pH reversed-phase chromatography with fraction concatenation for 2D proteomic analysis was performed as previously described[1] with the following changes. A Shimadzu Prominance HPLC (Shimadzu, Columbia, MD) with a Hot Sleeve-25L Column Heater (Analytical Sales & Products, Inc., Pompton Plains, NJ) was used with a Security Guard precolumn housing a Gemini NX C18 cartridge (Phenomemex, Torrance, CA) attached to a C18 XBridge column, 150 mm x 2.1 mm internal diameter, 5 µm particle size (Waters Corporation, Milford, MA). Buffer A was 20 mM ammonium formate, pH 10, in 98:2 water:acetonitrile and Buffer B was 20 mM ammonium formate, pH 10, in 10:90 water:acetonitrile. The flow rate was 200 µl/min with a gradient from 2-7% buffer B over 0.5 min, 7-15% buffer B over 7.5 min, 15-35% buffer B over 45 min, and 35-60% buffer B over 15 min. Fractions were collected every 2 minutes and UV absorbances were monitored at 215 nm and 280 nm. Peptide containing

fractions were divided into two equal numbered groups, “early” and “late”. A volume equal to 15 milli-absorbance units of the first “early” fraction was concatenated with the first “late” fraction, and so on. Concatenated samples were dried *in vacuo*, resuspended in load solvent (98:2:0.01, water:acetonitrile:formic acid) and run on the Thermo Orbitrap Fusion™ Tribrid™ mass spectrometer.

Orbitrap Fusion Liquid Chromatography - Mass Spectrometry (LC-MS) Peptide Analysis. Dried peptide fractions were reconstituted in 97.9:2:0.1, H2O:acetonitrile (ACN):formic acid (FA) and analyzed ~200 nanograms of each fraction by capillary LC-MS with a Thermo Fisher Scientific, Inc (Waltham, MA) Easy NanoLC 1100 system in-line with Orbitrap Fusion mass spectrometer (Thermo Fisher Scientific). Peptides were loaded directly on-column in solvent A (99.9:0.1, H2O:FA) at maximum pressure (800 bar). Peptides were then separated on a self-packed C18 column (Dr. Maisch GmbH ReproSil-PUR 1.9 um 120 Å C18aq, 100 µm ID x 50 cm length at 55 °C with a biphasic gradient from 5 to 22% solvent B in 45 minutes and 35% B at 70 minutes with a flowrate of 300 nl/minute. A top-12 data dependent acquisition method was employed with the following MS parameters: ESI voltage 2.1 kV, ion transfer tube 275 °C; Easy-IC internal calibration, Orbitrap MS1 scan 60k resolution in profile mode from 380 – 1580 *m/z* with 250 msec injection time, 250% (1E6) automatic gain control (AGC); MS2 triggered on the top 15 most abundant ions above 5E4 counts, 1.6 Da quadrupole isolation window, fixed HCD activation with 35% collision energy, Orbitrap detection with 60K resolution at 200 *m/z*, first mass fixed at 110 *m/z*, 250 msec max injection time, 5E4 AGC and 40 sec dynamic exclusion duration with +/- 10 ppm mass tolerance.

Database Searching. Peptide tandem MS were processed using Sequest (Thermo Fisher Scientific, San Jose, CA, in Proteome Discoverer 2.4). The rat (taxonID 10116) Universal Proteome (UP000002494) target protein sequence database was downloaded from UniProt (www.uniprot.org/) on Nov 21, 2019 and merged with a common lab contaminant protein database (http://www.thegpm.org/cRAP/index.html); the number of protein sequences was 29,996 sequences. The digestion enzyme trypsin, specific specificity with maximum 2 missed cleave sites; fragment ion mass tolerance was 0.10 Da and precursor tolerance was 120 ppm. The variable modifications were set for oxidation of methionine, pyroglutamic acid conversion from glutamine, deamidation of asparagine, acetyl of the protein N-terminus and TMT10plex of lysine and peptide N-terminus. Carbamidomethyl of cysteine was specified as a fixed modification.

Phosphorylated Peptide TMT™ Enrichment – The phosphopeptide TMT™ sample aliquot was dried down *in vacuo*. The sample was cleaned with a 1 mL Oasis HLB solid phase extraction cartridge (Waters Corporation, Milford, MA) and the eluate was dried *in vacuo*. The sample was phosphopeptide enriched with the High-Select™ TiO2 phosphopeptide kit and the High-Select™ Fe-NTA phosphopeptide kit using the High-Select™ SMOAC (Sequential enrichment of Metal Oxide Affinity Chromatography) protocol per the manufacturer’s instructions (ThermoFisher Scientific, Rockford, IL). The TiO2 and Fe-NTA samples were reconstituted and run separately on a Thermo Orbitrap Fusion™ Tribrid™ mass spectrometer.

Orbitrap Fusion LC - MS Phosphopeptide Analysis. Dried peptide fractions were reconstituted from the TiO2 and FeNTA phosphopeptide-enriched fractions in 97.9:2:0.1, H2O:acetonitrile (ACN):formic acid (FA) and analyzed 25% of each peptide pool by capillary LC-MS with a Thermo Fisher Scientific, Inc (Waltham, MA) Easy NanoLC 1100 system in-line with Orbitrap Fusion mass spectrometer (Thermo Fisher Scientific). Peptides were loaded directly on-column in solvent A (99.9:0.1, H2O:FA) at maximum pressure (800 bar). Peptides were then seperated on a self-packed C18 column, (Dr. Maisch GmbH ReproSil-PUR), 1.9 µm 120 Å C18aq , 100

µm ID x 50 cm length at 55 °C with a biphasic gradient from 5 to 22% solvent B in 75 minutes, and 35% B at 120 minutes with a flowrate of 400 nl/minute. A top-12 data dependent acquisition method was employed with the following MS parameters: ESI voltage 2.1 kV, ion transfer tube 275 °C; Easy-IC internal calibration, Orbitrap MS1 scan 60k resolution in profile mode from 380 – 1580 *m/z* with 250 msec injection time, 250% (1E6) automatic gain control (AGC); MS2 triggered on the top 12 most abundant ions above 5E4 counts, 2 Da quadrupole isolation window, fixed HCD activation with 35% collision energy, Orbitrap detection with 60K resolution at 200 *m/z*, first mass fixed at 110 *m/z*, 250 msec max injection time, 5E4 AGC and 40 sec dynamic exclusion duration with +/- 10 ppm mass tolerance.

Database searching. Peptide tandem MS was processed using Sequest (Thermo Fisher Scientific, San Jose, CA, in Proteome Discoverer 2.4). The rat (taxonID 10116) Universal Proteome (UP000002494) target protein sequence database was downloaded from UniProt (www.uniprot.org/) on Nov 21, 2019 and merged with a common lab contaminant protein database (http://www.thegpm.org/cRAP/index.html); the number of protein sequences was 29,996 sequences. The digestion enzyme trypsin, specific specificity with maximum 2 missed cleave sites; fragment ion mass tolerance was 0.10 Da and precursor tolerance was 120 ppm. The variable modifications were set for phosphorylation of serine, threonine and tyrosine, oxidation of methionine, pyroglutamic acid conversion from glutamine, deamidation of asparagine, acetyl of the protein N-terminus and TMT10plex of lysine and peptide N-terminus. Carbamidomethyl of cysteine was specified as a fixed modification.

Validation by immunoblotting

Western blot was performed to validate some of the proteomic and p-proteomic findings. 20 µg of protein lysate, from left ventricular myocardium, was loaded and electrophoresed using SDS-PAGE gel then transferred to a PVDF membrane. The membrane was blocked for 1 hr with a blocking solution (0.15 mol/L sodium chloride, 3 mmol/L potassium chloride, 25 mmol/L tris-base, 5% skim milk, and 0.05% tween-20). Note 5% bovine serum albumin (BSA) was used for phospho-antibodies (Ab). Blots were incubated with the primary Ab overnight at 4ºC. The following primary antibodies were used: GAPDH, p-T172-AMPK, p-S485/491-AMPK, AMPK, p-T286-CaMKII, CaMKII, p-S21-GSK3α, p-S9-GSK3β, GSK3α, GSK3β, p-S23/24-TNNI3, TNNI3 and PP1α (Cell signaling, Danvers, MA, USA 1:1000 dilution), rodent ETC complexes cocktail, HADHA, HADHB and MCAD (Abcam Cambridge, MA, USA, 1/2500 dilution), PGC-1α (Novus Biologicals, Littleton, CO, USA 1:2500 dilution) and PLN and p-S16-PLN (Badrilla Ltd, Leeds, LS2 9DF, UK, 1:3000 dilution). The second day, after three washings with TBS-0.05% Tween-20, blots were incubated with secondary horseradish peroxidase conjugated antibody (Thermo Scientific, Barrington, IL, USA 1:10000 dilution) for 45 min. The blot was washed three times with TBS-0.05% Tween-20 and then a supersignal west pico chemiluminescent substrate (Thermo Scientific, Barrington, IL, USA) was used for detection of protein bands using a ChemiDoc XRS+ imaging system (Bio-Rad, Hercules, CA, USA). Band densities were quantified using image lab software (Bio-Rad, Hercules, CA, USA) and were normalized to GAPDH band densities, from the same blot that was used for specific protein detection, to correct for variations in protein loading. The p-protein band densities were normalized to total protein band densities obtained from the same blot.

Discovery based non-targeted Central Carbon Metabolism and Acyl-Carnitine analysis by LC-MS.

Sample Preparation. Cardiac muscle samples were carefully weighed (50 mg ± 2 mg) into microtubes and 100 µL of water was added to each sample. The samples were probe-sonicated

at 40% power for 3-6 seconds and placed on wet ice. 500 µL of 8:1:1, Methanol:Chloroform: Water, containing a mixture of ^{13}C–labeled Glycolysis/TCA and Acyl-carnitine internal standards was added. Microtubes were vortexed, and allowed to incubate at 4 °C for 10 minutes to complete metabolite extraction. Samples we vortexed a second time, and then centrifuged at 14,000 RPM for 10 min at 4°C. Pools were created for each separate analysis by removing 10 µL of each sample and combining into an autosampler vial; this was treated like the other samples for the remainder of the analysis. For the samples, 200 µL of the extraction solvent was transferred to separate autosampler vials for Central Carbon and Acyl-carnitine analysis, then dried at ambient temperature under a stream of nitrogen for 1 hr. Prior to analysis, samples were reconstituted in 100 µL of a 8:2, Water:Methanol mix and vortexed to aid resuspension.

<u>Central Carbon Metabolism Ion Pairing Reverse Phase LC-MS Analysis.</u> Analysis was performed on an Agilent system consisting of an Infinity Lab II UPLC coupled with a 6545 Quadruole Time-of-flight mass spectrometer (Agilent Technologies, Santa Clara, CA) using a JetStream ESI source in negative mode. The following source parameters were used: Gas Temp: 250°C, Gas Flow: 13 L/min, Nebulizer: 35 psi, Sheath Gas Temp: 325°C, Sheath Gas Flow: 12 L/min, Capillary: 3500 V, Nozzle Voltage: 1500 V. The UPLC was equipped with a 10-port valve configured to allow the column to be either eluted to the mass spectrometer or back-flushed to waste. The chromatographic separation was performed on an Agilent ZORBAX RRHD Extend 80Å C18, 2.1 × 150 mm, 1.8 µm column with an Agilent ZORBAX SB-C8, 2.1 mm × 30 mm, 3.5 µm guard column. The column temperature was 35°C. Mobile phase A consisted of 97:3, Water:Methanol and mobile phase B was 100% methanol; both A and B contained tributylamine and glacial acetic acid at concentrations of 10mM and 15mM, respectively. The column was back-flushed with mobile phase C (100% acetonitrile, no additives) between injections for column cleaning. The LC gradient was as follows: 0-2 min, 0% B; 2-12 min, linear ramp to 99% B; 12-17.5 min, 99% B. At 17.5 minutes, the 10-port valve was switched to reverse flow (back-flush) through the column, and the solvent composition changed to 99% C. From 20.5-21 min the flow rate was ramped to 0.8 mL/min, held until 22.5 min, and then reduced to 0.6mL/min. From 22.7-23.5 min the solvent was ramped from 99% to 0% C, while flow was simultaneously ramped down from 0.6-0.4mL/min and held until 29.4 min. At that point flow rate was returned to starting conditions at 0.25mL/min. The 10-port valve was returned to restore forward flow through the column at 28.5 min. An isocratic pump was used to introduce reference mass solution through the reference nebulizer for dynamic mass correction. Total run time was 30 min. The injection volume was 5 µL.

<u>Acyl-Carnitine LC-MS analysis.</u> Analysis was performed on an Agilent system consisting of a 1290 UPLC module coupled with a 6490 Triple Quadrupole mass spectrometer (Agilent Technologies, CA, USA.) A 1 µL injection of acyl carnitine metabolites were separated on an Acquity HSS-T3 1.8 µM, 2.1 x 50 mm column (Waters, Milford, MA) maintained at 40°C, using 10 mM ammonium acetate in water, adjusted to pH 9.9 with ammonium hydroxide, as mobile phase A, and acetonitrile as mobile phase B. The flow rate was 0.25 mL/min and the gradient was linear 0% to 80% A over 7 min, then 80 to 100% over 1.5 min, followed by isocratic elution at 100% A for 5 min. The system was returned to starting conditions for 3 min to allow for column re-equilibration before injecting another sample. The mass spectrometer was operated in ESI- mode with the following instrument settings: Gas temp: 275°C, flow: 15 L/min, nebulizer: 35 psi, capillary 3500 V, sheath gas 250°C, and sheath gas flow 11 L/min. The ion funnel high/low pressure RF settings were 150/60 V, respectively. Acyl-carnitine transitions were monitored for the 85 Da product ion that is common to each carnitine species.

Table S1. Echocardiographic parameters of studied animals in Sham, MOD and SHF groups.

Groups / Parameters	Week 3 post-AAB (Concentric hypertrophy)				Week 8 post-AAB (Phenotype development)		
	**Sham n = 3**	**MOD n = 3**	**SHF n = 3**		**Sham n = 3**	**MOD n = 3**	**SHF n = 3**
BW (g)	323 ± 8	313 ± 2	293 ± 16		496 ± 10‡	495 ± 31‡	502 ± 9‡
IVSd (cm)	0.17 ± 0.01	0.26 ± 0.02*	0.26 ± 0.01*		0.19 ± 0.01	0.29 ± 0.01*	0.27 ± 0.01*
LVPWd (cm)	0.17 ± 0.01	0.26 ± 0.02*	0.26 ± 0.01*		0.21 ± 0.02	0.30 ± 0.01*	0.28 ± 0.01*
LVIDd (cm)	0.68 ± 0.06	0.60 ± 0.03	0.58 ± 0.04		0.69 ± 0.03	0.70 ± 0.04	0.83 ± 0.03*†‡
LVIDs cm)	0.26 ± 0.04	0.15 ± 0.04*	0.14 ± 0.03*		0.28 ± 0.02	0.23 ± 0.03	0.47 ± 0.01*†‡
LVEDV (µl)	472 ± 36	342 ± 63	352 ± 45		561 ± 71	620 ± 32‡	901 ± 116*†‡
LVESV (µl)	90 ± 15	26 ± 7.5*	40 ± 10*		102 ± 22	125 ± 8‡	432 ± 63*†‡
LVEF (%)	81 ± 1.8	92 ± 1*	89 ± 1.4*		82 ± 1.9	80 ± 1.2‡	52 ± 1.0*†‡
HW/BW (mg/g)					2.35 ± 0.13	4.04 ± 0.31*	4.67 ± 0.16*†
LVW/BW (mg/g)					1.66 ± 0.14	3.06 ± 0.22*	3.17 ± 0.04*
RVW/BW (mg/g)					0.41 ± 0.03	0.50 ± 0.04	0.85 ± 0.08*†

Data are presented as mean ± standard deviation. Statistical analysis was performed in Prism software version 9.1.0 using one-way ANONA with Benjamini correction method. A p-value of < 0.05 was considered significant.

Abbreviations: IVSd: Interventricular septal diameter, LVPWd: left ventricular (LV) posterior wall diameter, LVIDd: LV end-diastolic diameter, LVIDs: LV end-systolic diameter, LVEDV: LV end-diastolic volume, LVESV: LV end-systolic volume, LVEF: LV ejection fraction, BW: body weight, HW: heart weight, LVW: LV weight, and RVW: right ventricular weight

*P < 0.05 vs Sham
†P < 0.05 vs MOD
‡P < 0.05 vs Week 3

Supplemental figures and figure legends

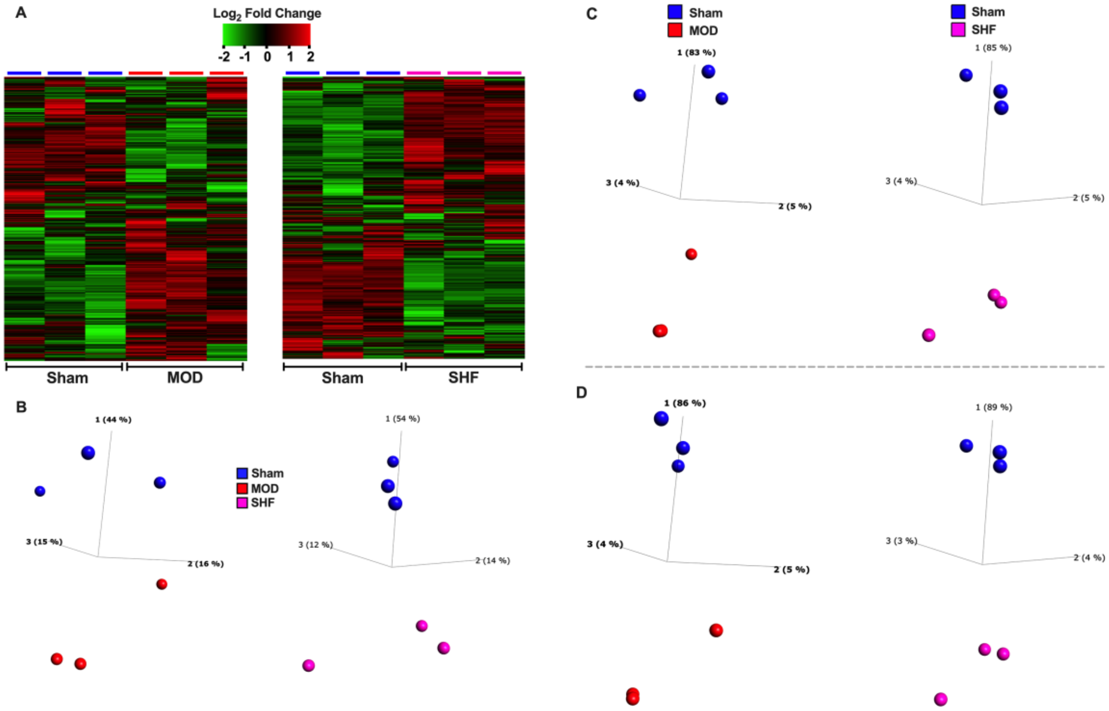

Figure S1. Heat maps (**A**) and PCA plots (**B**) of all identified proteins in the MOD and SHF proteomic runs. The heat maps are showing the differential $\log_2$ fold change of all identified proteins in Sham, MOD and SHF groups. The PCA plots indicate the variance in the MOD and SHF biological samples relative to Sham; suggesting a significant biological phenotypic changes in the MOD and SHF groups relative to Sham. PCA plots for the identified proteins (**C**) that changed in MOD (left) and SHF (right) relative to Sham and for proteins related to metabolism and respiration (**D**) that changed in MOD (left) and SHF (right) relative to Sham. Data analysis in C-D was performed in Qlucore bioinformatics software. Heat maps (**E**), PCA plots (**F**), and Venn diagrams (**G**) for the proteins that changed in MOD (left) and SHF (right) relative to Sham. Data analysis in E-G was performed in Scaffold bioinformatics software. Qlucore bioinformatics software was used to generate heat maps, PCA

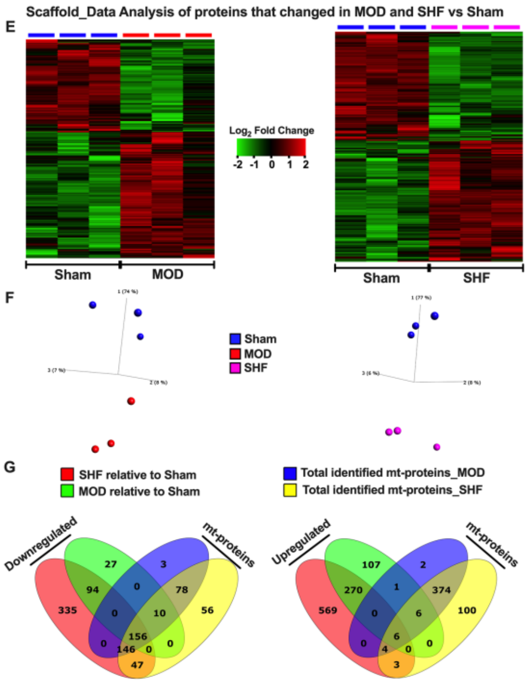

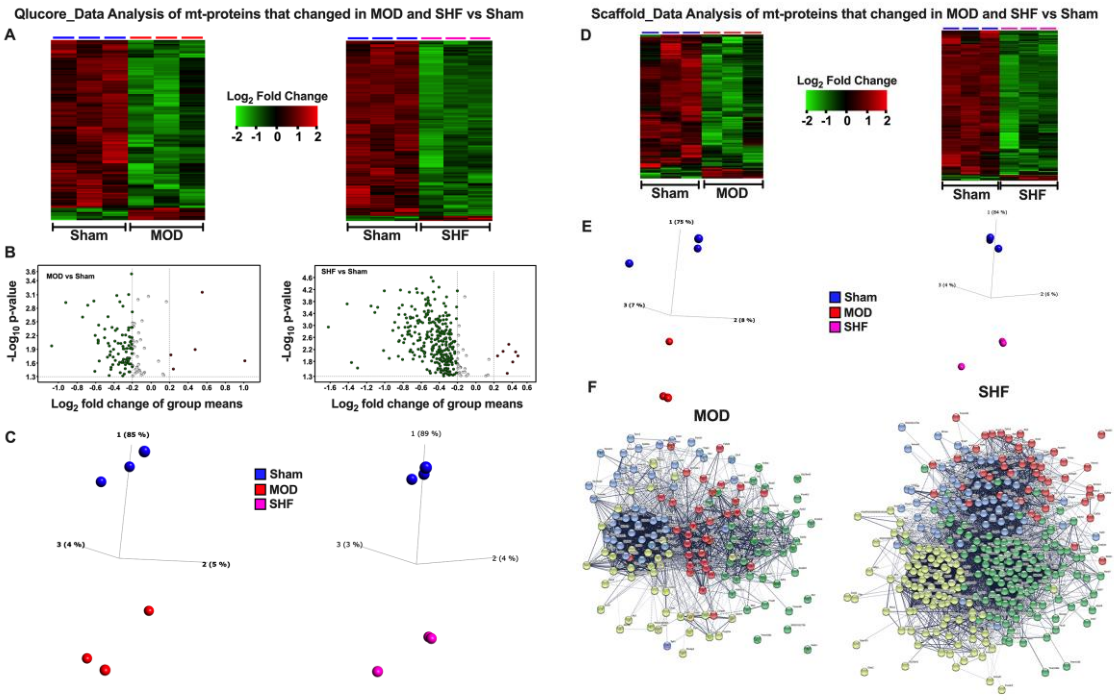

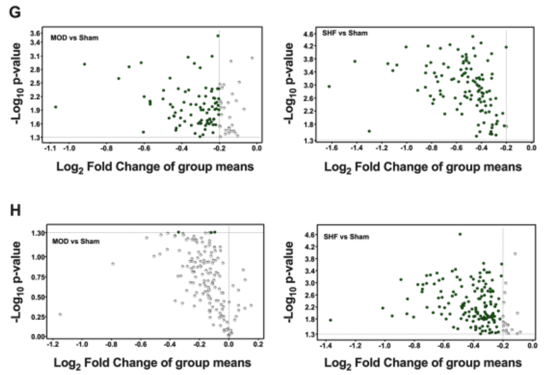

Figure S2. Heat maps (**A**), Volcano plots (**B**), and PCA plots (**C**) of mt-proteins that changed in MOD (left) and SHF (right) relative to Sham. Data analysis in A-C was performed in Qlucore bioinformatics software. Heat maps (**D**) and PCA plots (**E**) of mt-proteins that changed in MOD (left) and SHF (right) relative to Sham. Data analysis in D-E was performed in Scaffold bioinformatics software. Qlucore bioinformatics software was used to generate heat maps and PCA plots. **F.** Protein-protein interaction (PPI) network of mt-proteins that decreased in MOD (left) and SHF (right) relative to Sham was generated using STRING database, PPI enrichment p-value of 1.0e-16. Each node represents a protein, whilst edges (lines) represent protein-protein associations based on physical and physiological interaction by confidence. The thicker the line is, the higher is the confidence. **G.** Volcano plots of the commonly identified, 114, mt-proteins that decreased in MOD (left) and SHF (right) relative to Sham. **H.** Volcano plots of the commonly identified, 116, mt-proteins in MOD and SHF proteomic runs that decreased in SHF only relative to Sham.

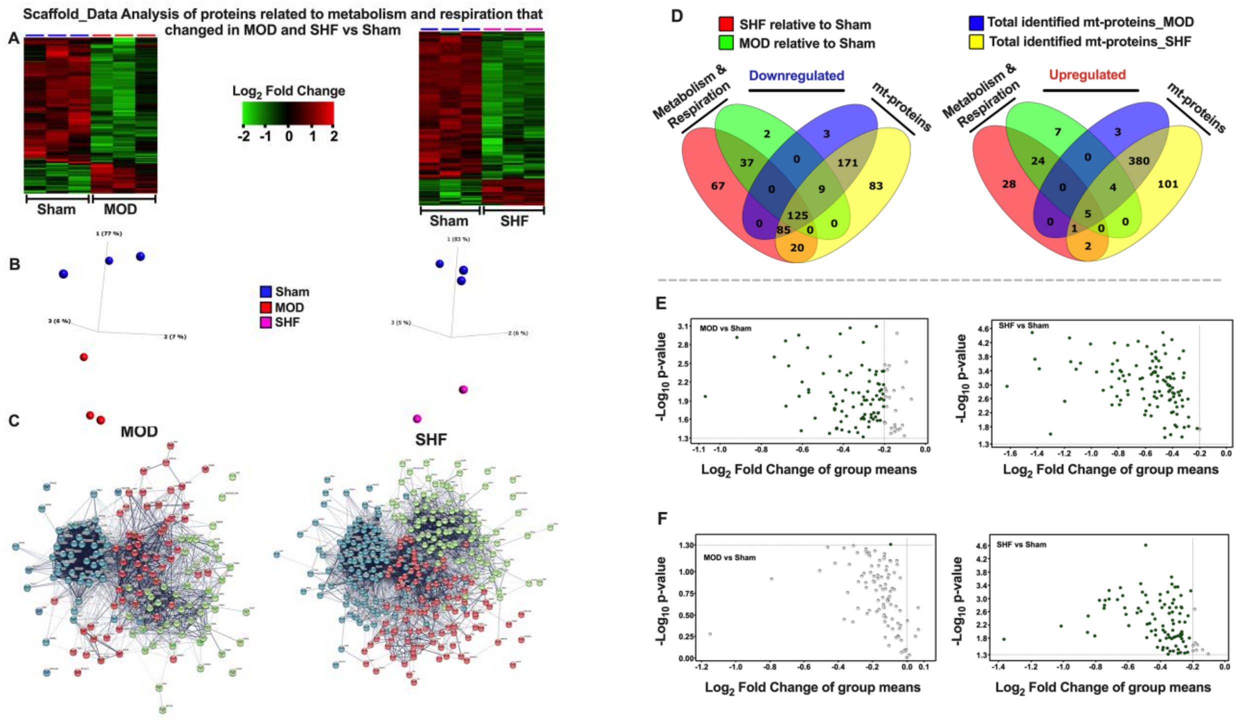

Figure S3. Heat maps (**A**) and PCA plots (**B**) of proteins related to metabolism and respiration that changed in MOD (left) and SHF (right) relative to Sham. Data analysis in A-B was performed in Scaffold bioinformatics software. Qlucore bioinformatics software was used to generate heat maps and PCA plots. **C.** PPI network for proteins related to metabolism and respiration that decreased in MOD (left) and SHF (right) relative to Sham was generated using STRING database, PPI enrichment p-value of 1.0e-16. Each node represents a protein, whilst edges (lines) represent protein-protein associations based on physical and physiological interaction by confidence. The thicker the line is, the higher is the confidence. **D.** Venn diagrams showing the number of identified proteins related to metabolism and respiration that decreased (left) or increased (right) in MOD and SHF groups compared to Sham, green and red diagrams, respectively. It also shows how many of those were mt-proteins (intersection of green and blue diagrams for MOD and red and yellow diagrams for SHF) vs non-mitochondrial proteins (numbers that fall outside the area of intersection). Data in A-B and D were analyzed by Scaffold bioinformatics software and Qlucore bioinformatics software was used for data presentation. **E.** Volcano plots of the commonly identified, 113, proteins related to metabolism and respiration that decreased in MOD (left) and SHF (right) relative to Sham. **H.** Volcano plots of the commonly identified, 100, mt-proteins related to metabolism and respiration in MOD and SHF proteomic runs that decreased in SHF only relative to Sham.

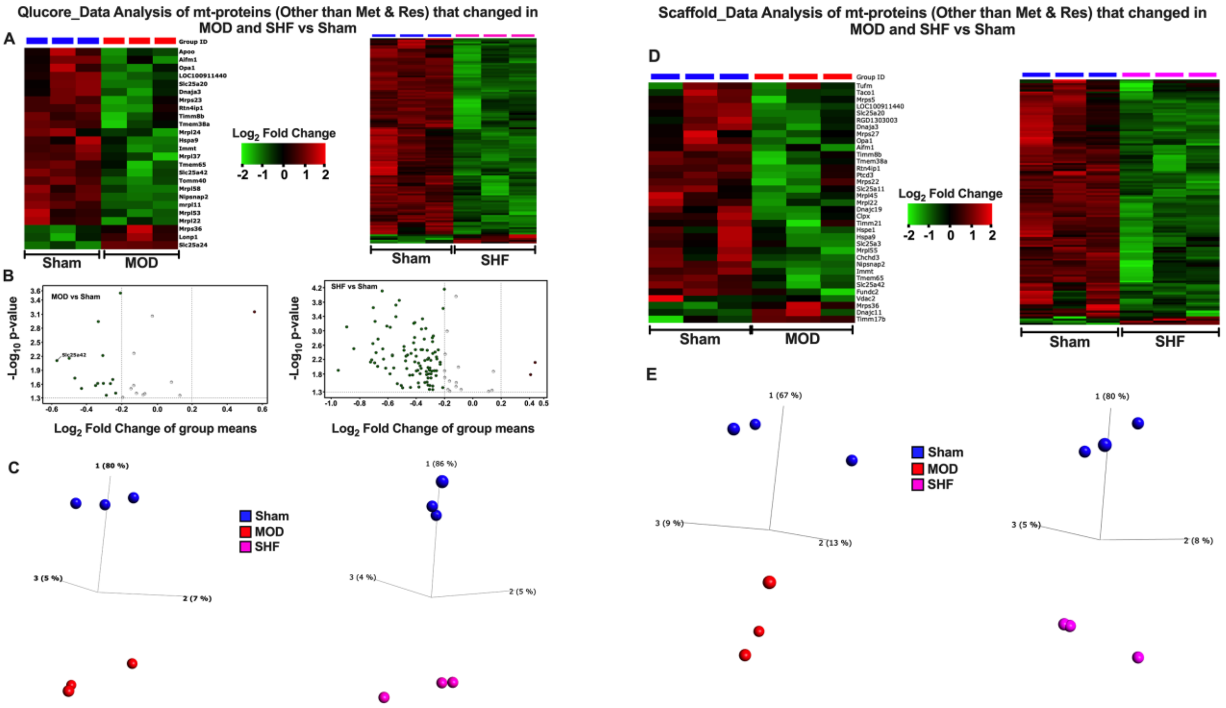

Figure S4. Heat maps (**A**), Volcano plots (**B**), and PCA plots (**C**) of mt-proteins, other than those involved in metabolism and respiration, that changed in MOD (left) and SHF (right) relative to Sham. Data analysis in A-C was performed in Qlucore bioinformatics software. Heat maps (**D**) and PCA plots (**E**) of mt-other proteins that changed in MOD (left) and SHF (right) relative to Sham. Data analysis in D-E was performed in Scaffold bioinformatics software. Qlucore bioinformatics software was used to generate heat maps and PCA plots.

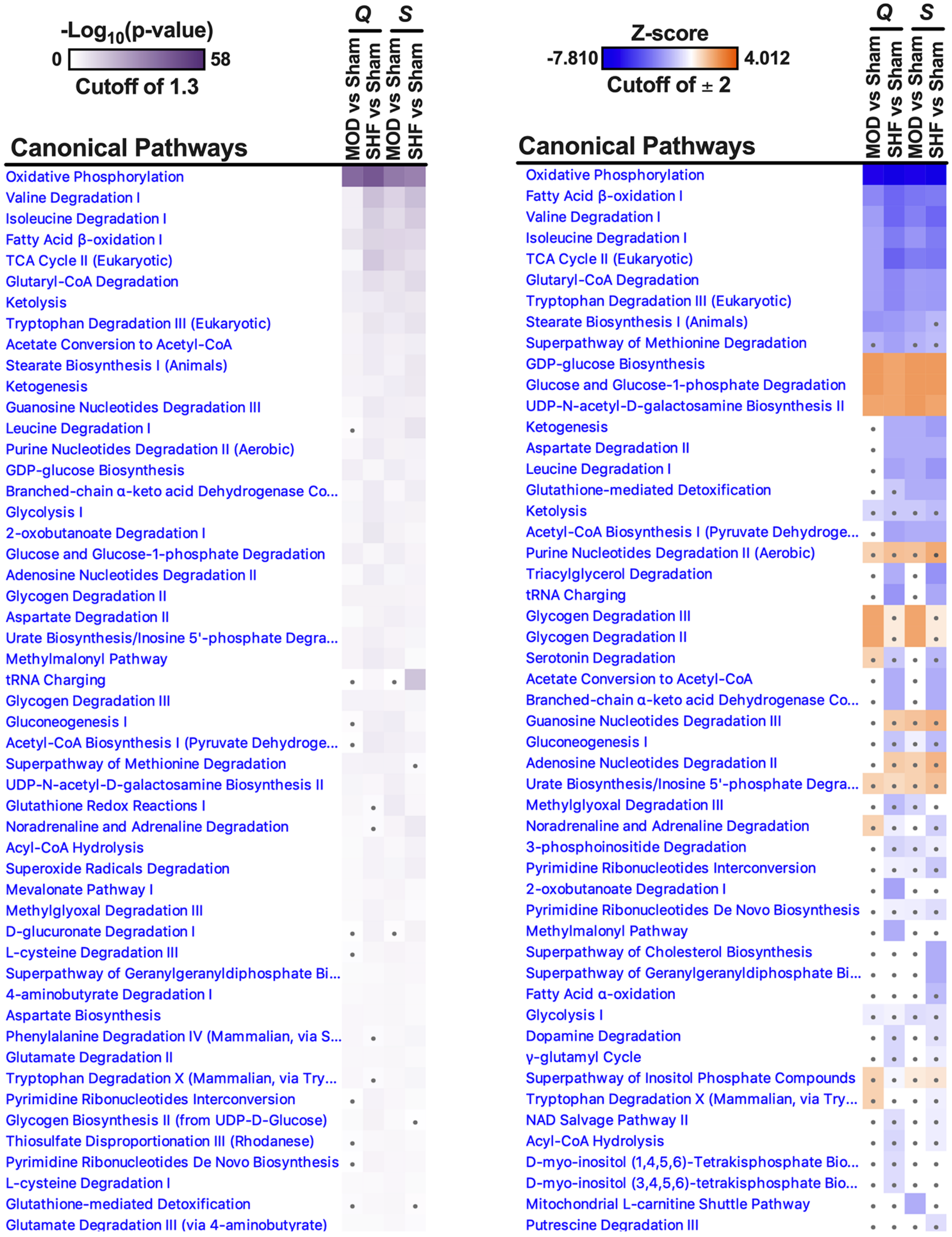

Figure S5. Comparison analysis performed in IPA showing heat maps of metabolic pathways that were enriched in the proteomic datasets in MOD and SHF relative to Sham. Heat maps presented by p-value and z-score. Metabolic pathways that were below the cutoff p-value or z-score are represented by a dot on the heat map.

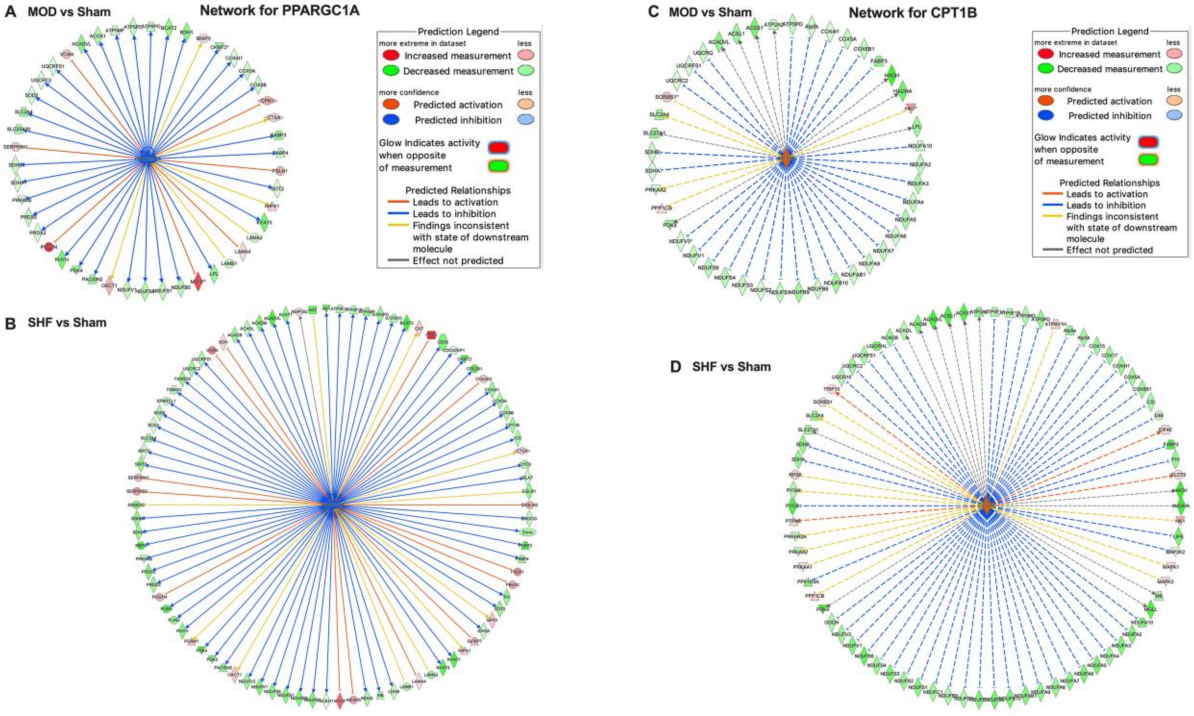

Figure S6. PPARGC1A network of proteins that changed in MOD (**A**) and SHF (**B**) relative to Sham. **C-D.** CPT1B associated network of proteins that changed in MOD and SHF relative to Sham. Please refer to Prediction legend for details.

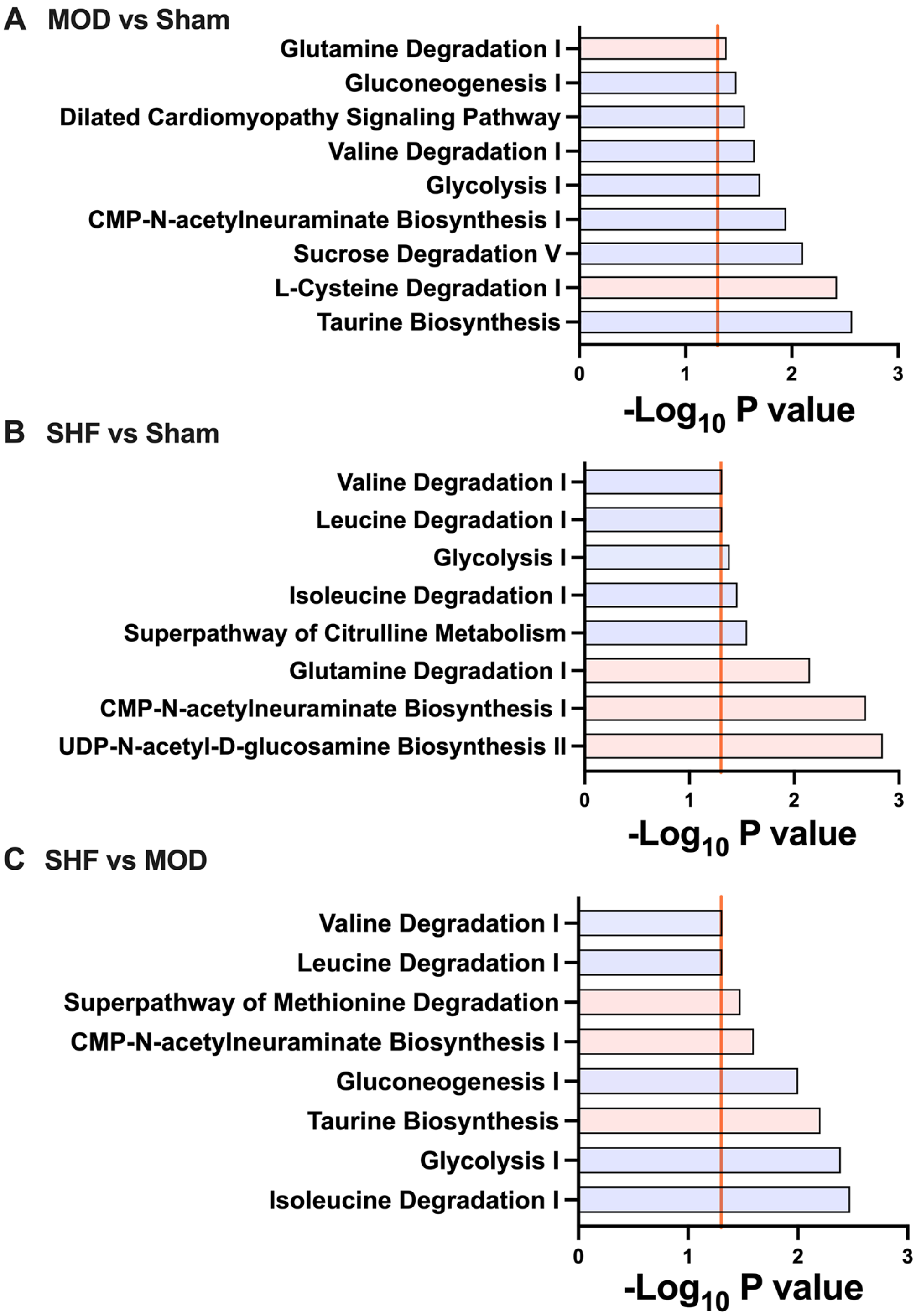

Figure S7. Metabolic pathways that changed in MOD (**A**) and SHF (**B**) relative to Sham, and in SHF relative to MOD (**C**) based on IPA Core Analysis of the metabolomics datasets using a cutoff p-value of < 0.05. Bar graphs are showing metabolic pathways that were enriched by p-value, presented as $-\text{Log}_{10}$ (p value) on the y-axis with predicted activation (orange) or inhibition (blue) for each of the metabolic pathways. A $-\text{Log}_{10}$ p-value cutoff of 1.3 and higher was considered significant.

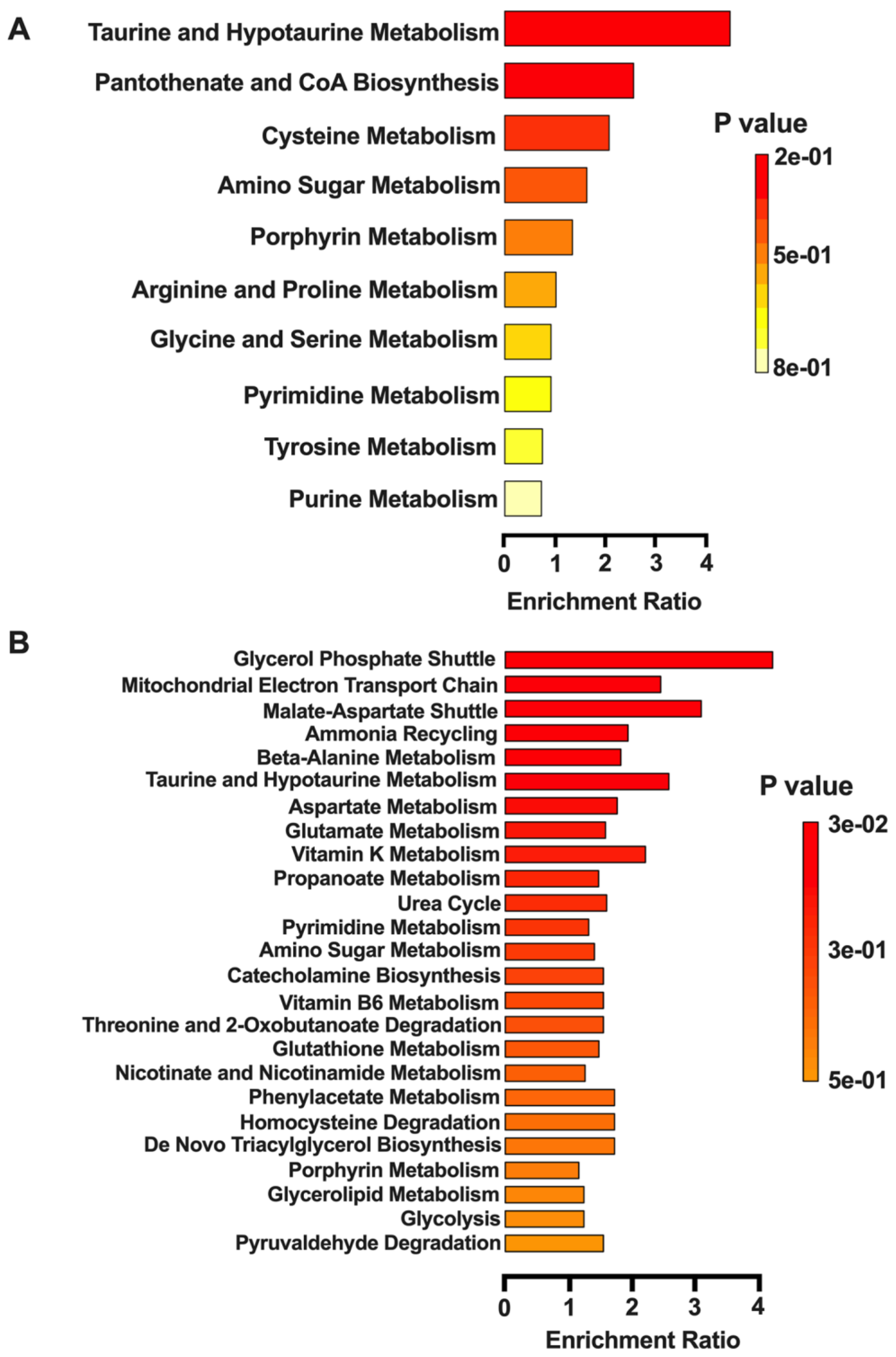

Figure S8. Enrichment pathway analysis, generated in Metaboanalyst bioinformatics software, showing the enrichment ratio for the metabolites that changed in MOD (**A**) and SHF (**B**) relative to Sham.

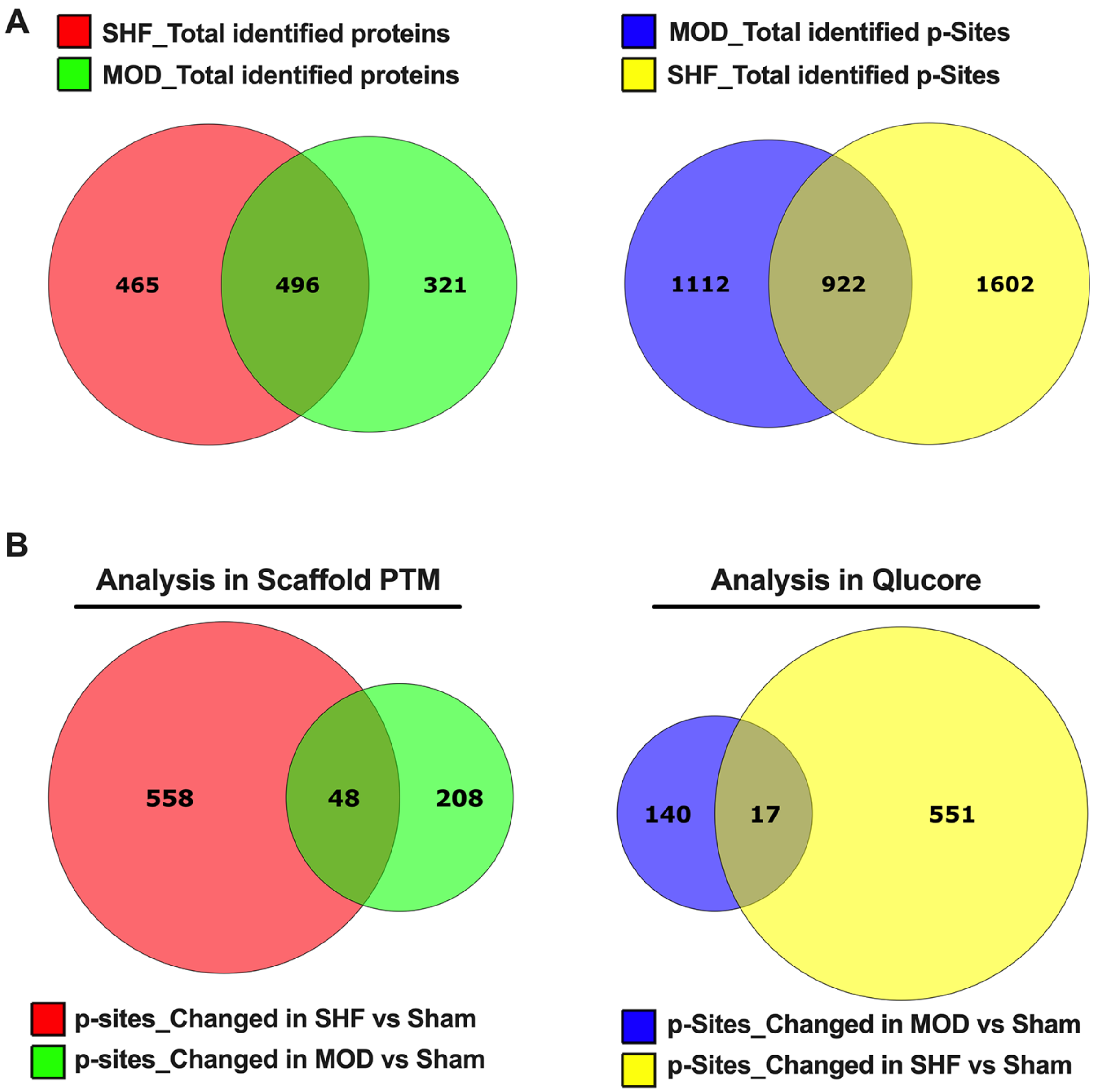

Figure S9. A. Venn diagrams showing the number of total identified proteins and phospho (p)-sites in MOD (green and blue circles, respectively) and SHF (red and yellow circles, respectively) p-proteomic runs. **B.** Venn diagrams showing the number of p-sites that changed in MOD and SHF analyzed by Scaffold PTM (left) and Qlucore (right) bioinformatics software.

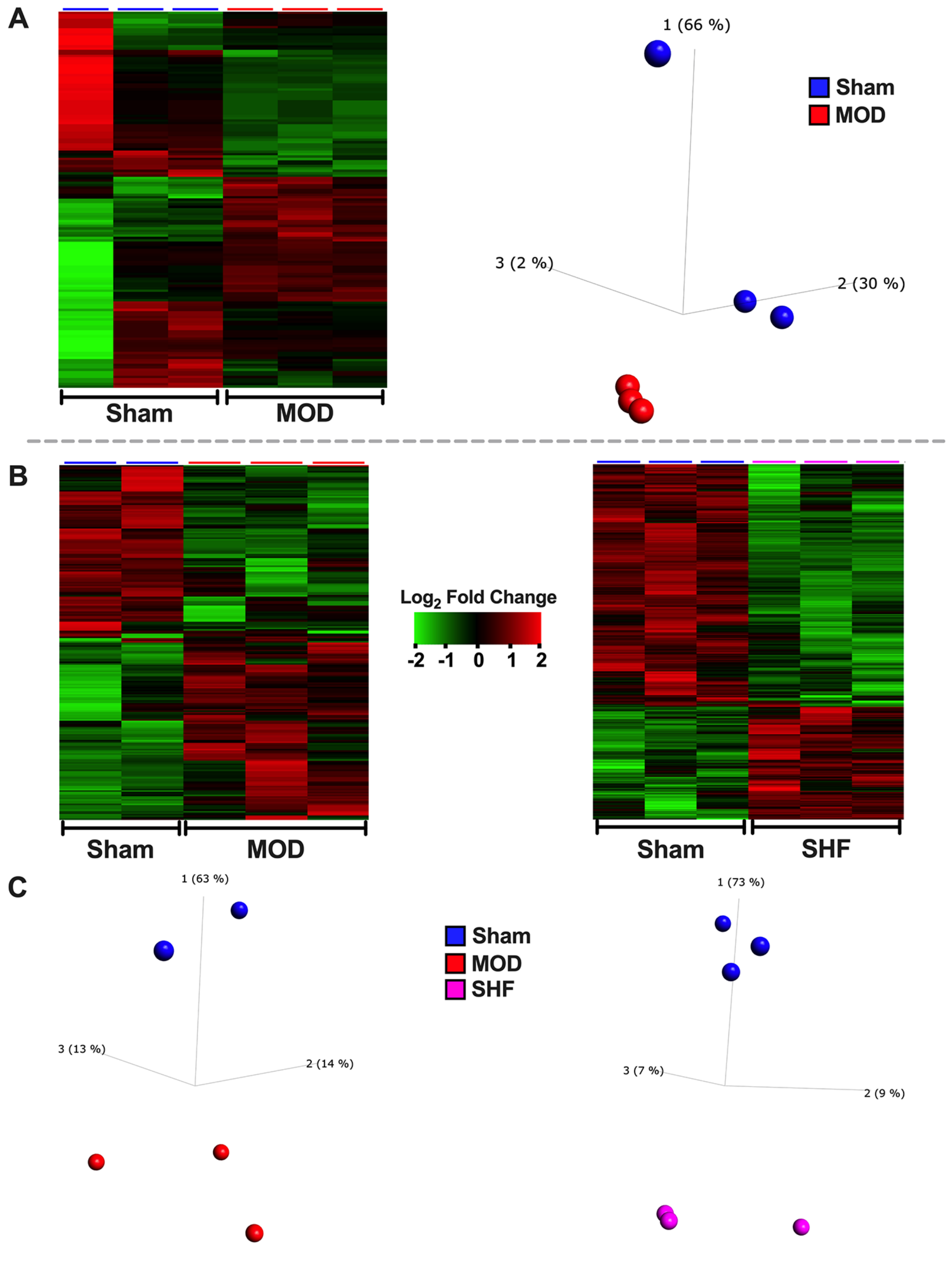

Figure S10. A. Heat map and PCA plot of the p-sites that changed in MOD relative to Sham. The heat map shows the Sham biological sample (first column of the heat map) that was an outlier and was eliminated. **B.** Heat maps and PCA plots of the p-sites that changed in MOD (left) and SHF (right) relative to Sham. Data were analyzed by Scaffold PTM bioinformatics software and Qlucore bioinformatics software was used to generate heat maps and PCA plots.

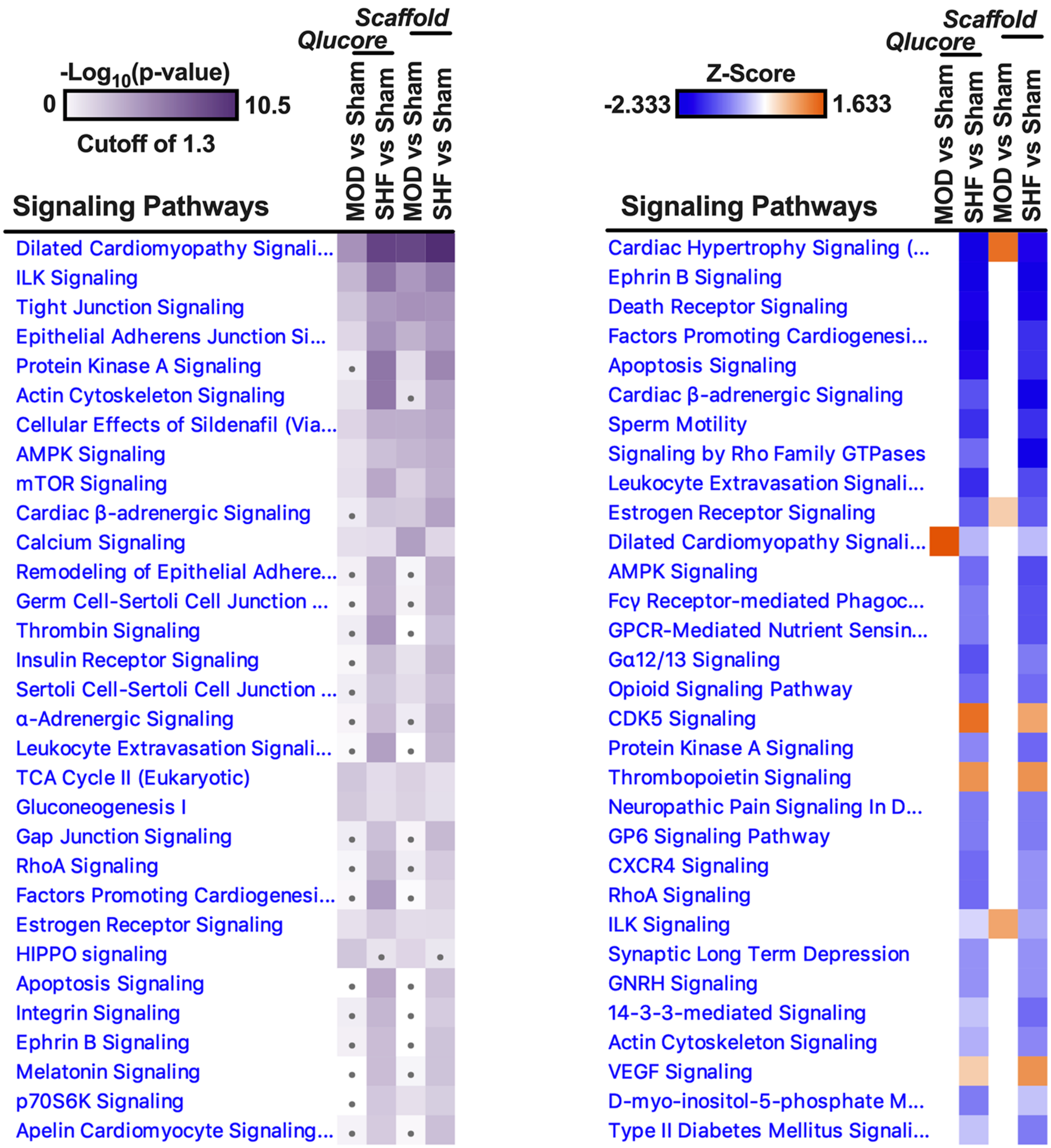

Figure S11. Signaling pathways that changed in MOD and SHF relative to Sham. Heat maps, generated in IPA using the Comparison Analysis" functionality of the analyzed p-proteomics datasets, show the signaling pathways that were enriched in MOD and SHF relative to Sham by p-value and z-score.

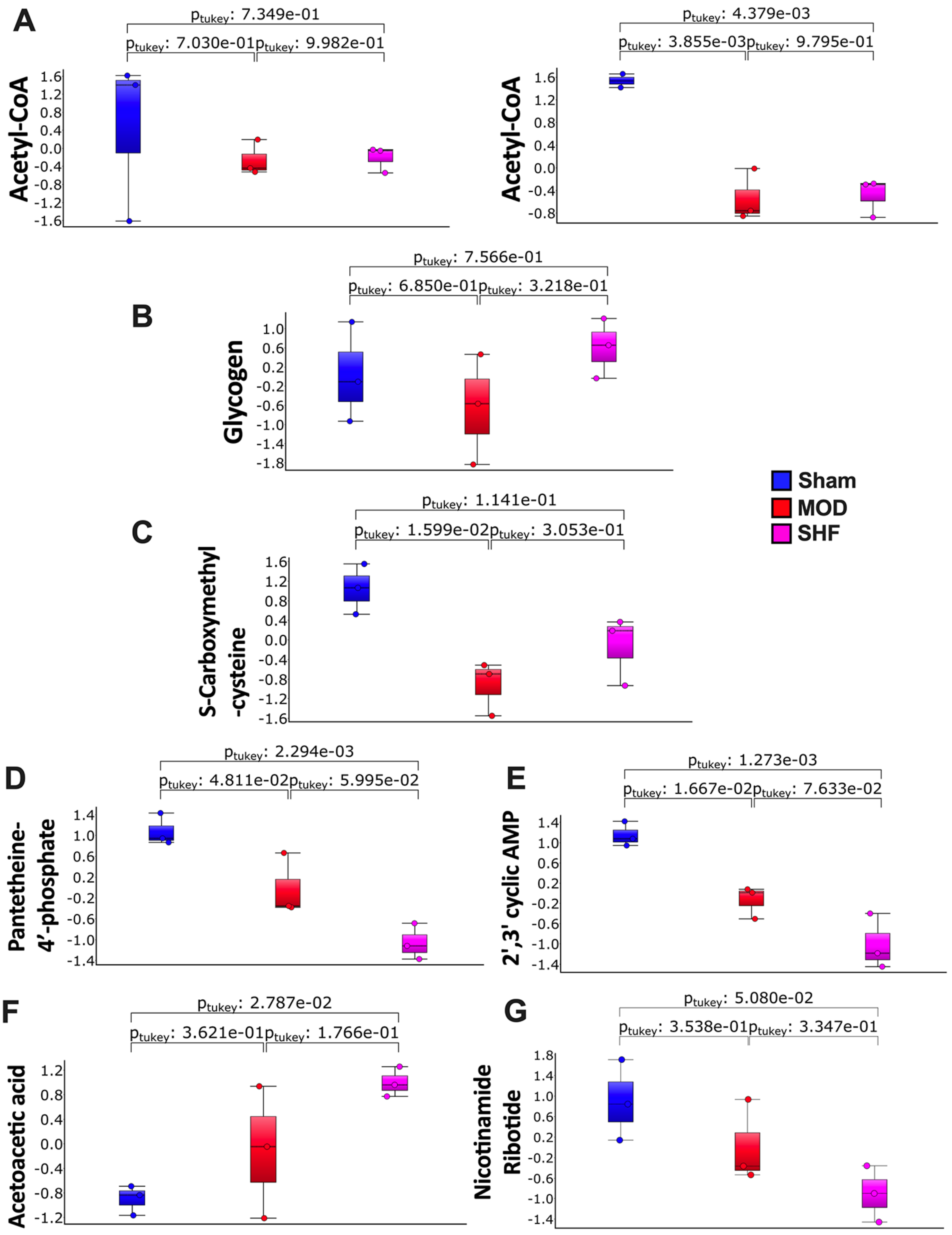

Figure S12. Box and Whisker plots showing the log_2 fold relative expression (minimum, median, interquartile, and maximum range) in Sham, MOD and SHF groups for the metabolites presented in A-G.

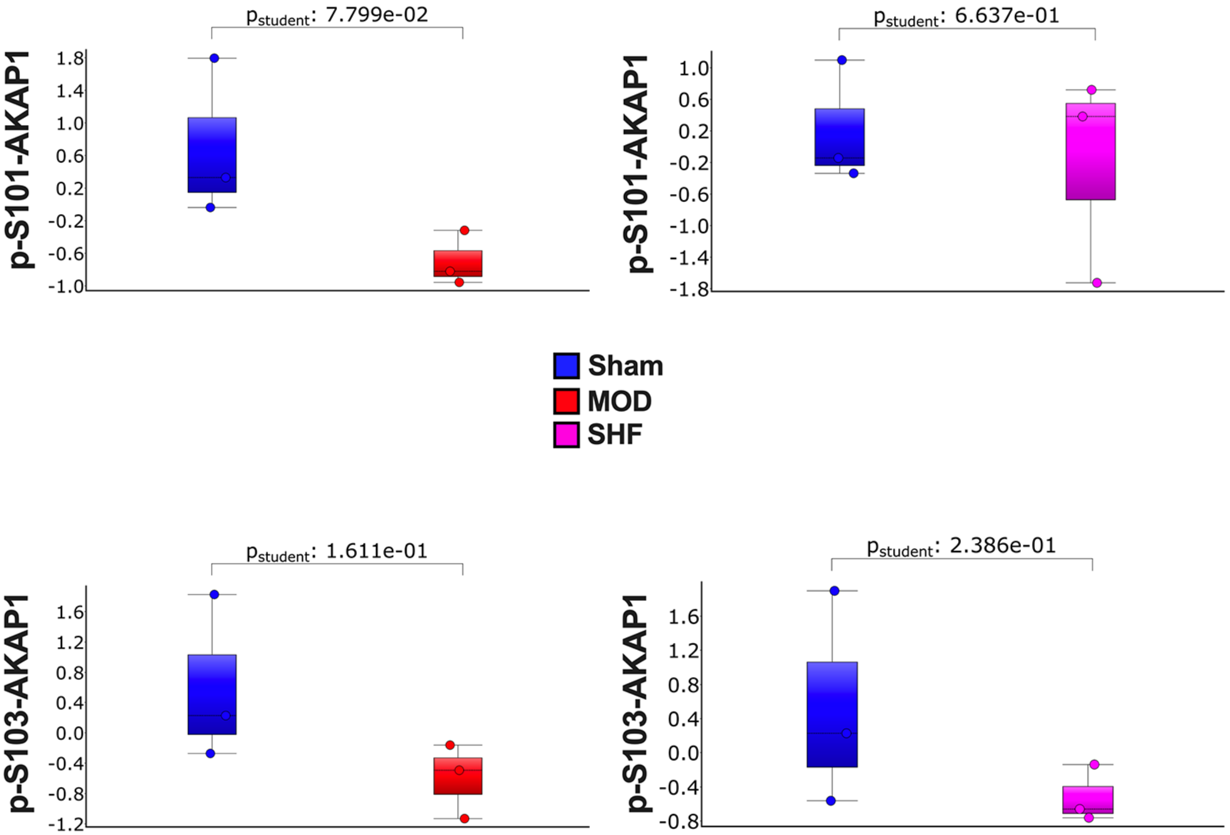

Figure S13. Box and Whisker plots showing the $\log_2$ fold relative expression (minimum, median, interquartile, and maximum range) for the A-kinase anchoring protein 1 (AKAP1) Serine 101 (p-S101-AKAP1) and Serine 103 (p-S103-AKAP1) residues in Sham, MOD and SHF groups.

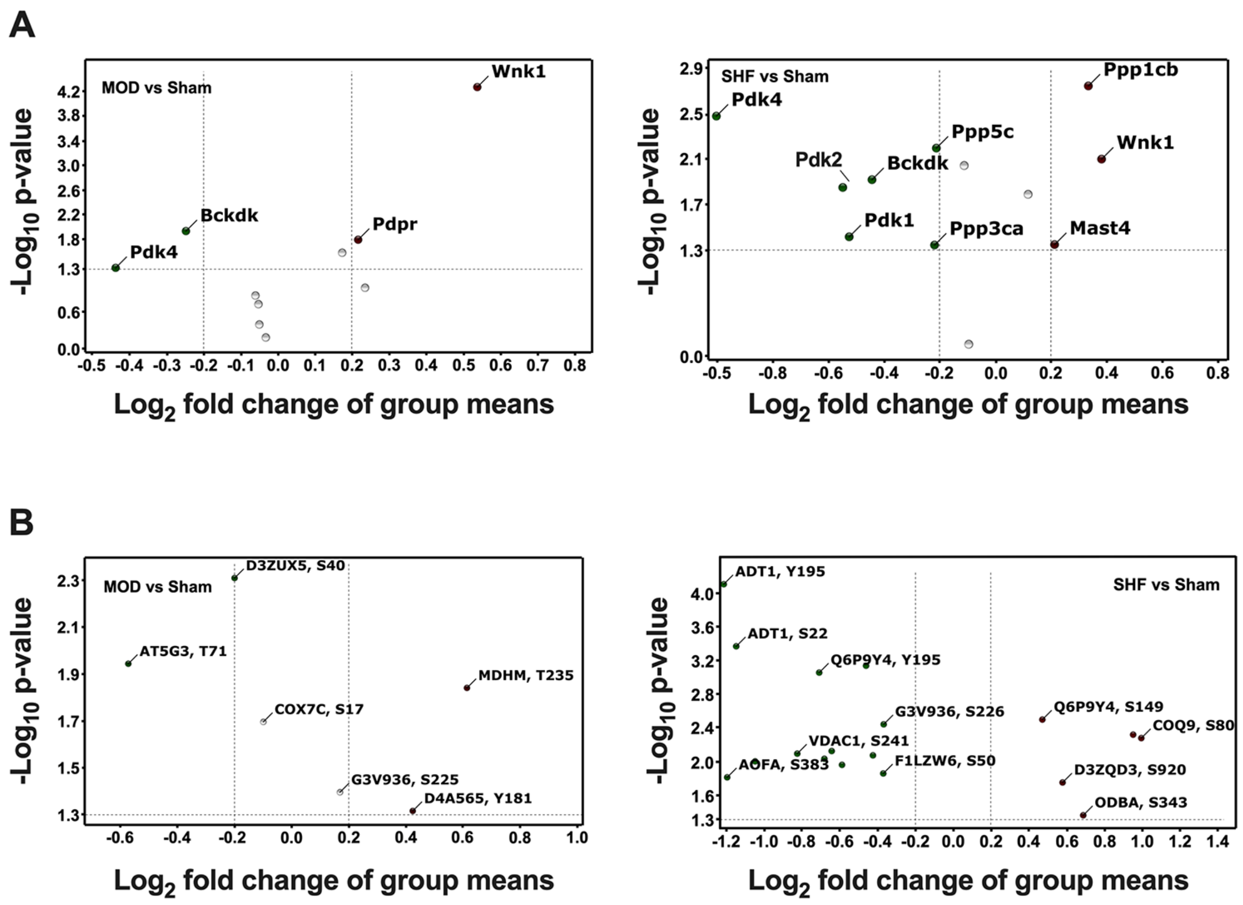

Figure S14. A. Volcano plots showing the $\log_2$ fold change of group means for the Serine/threonine kinases and phosphatases that changed in MOD (right) and SHF (left) relative to Sham. **B.** Volcano plots showing the $\log_2$ fold change of group means for the mt-protein p-sites that changed in MOD (left) and SHF (right) relative to Sham. Data in A and B were analyzed by Qlucore bioinformatics software and are presented in supplemental excel file 1 and 3, respectively. Please refer to excel file 1 and 3 for the name of the presented gene IDs in A and B, respectively.

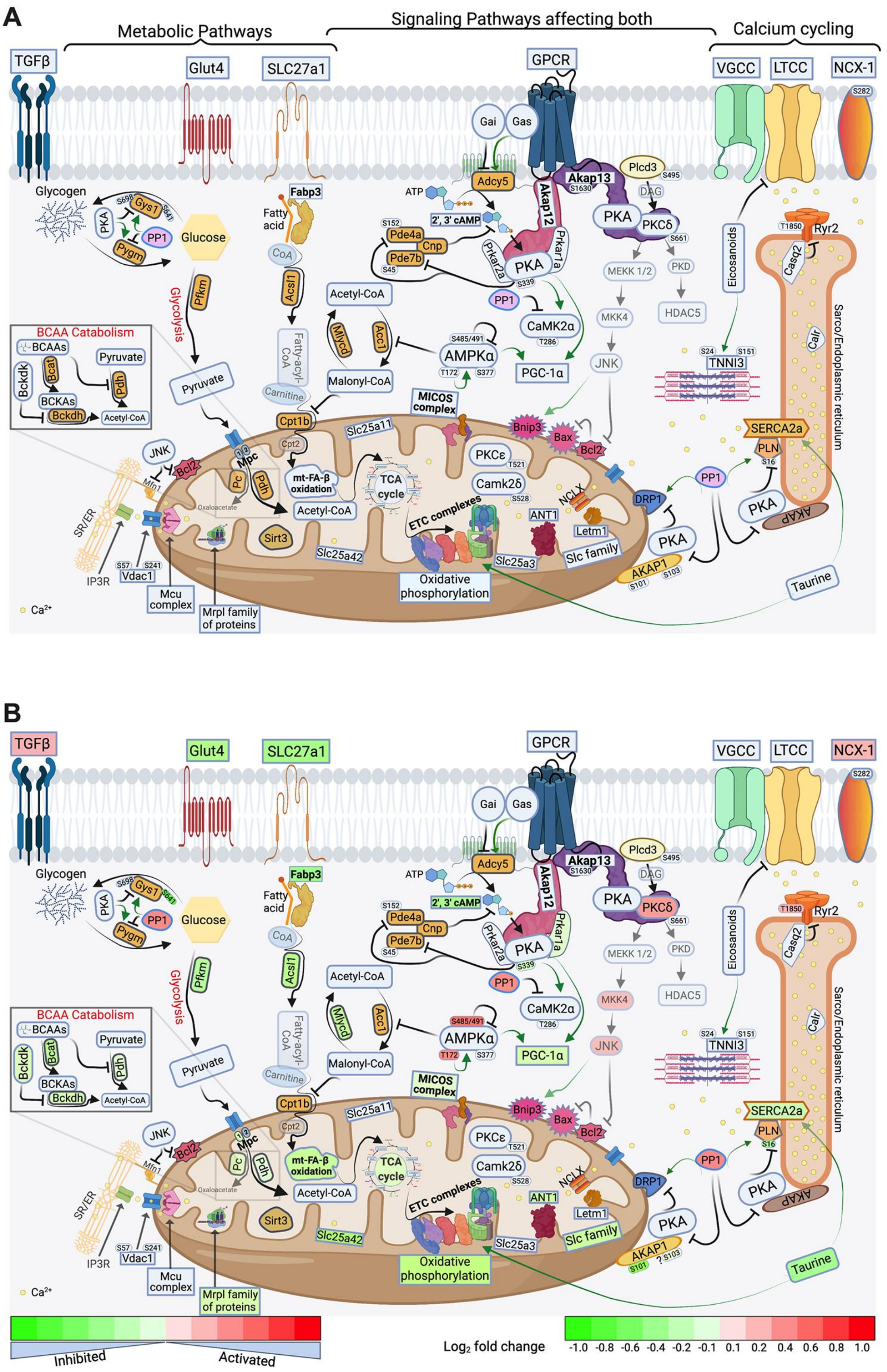
A
Metabolic Pathways
Signaling Pathways affecting both
Calcium cycling
TGFβ
Glut4
SLC27a1
GPCR
VGCC
LTCC
NCX-1
Gai
Gas
Adcy5
Akap13
Akap12
Plcd3
DAG
PKA
PKCδ
ATP
2', 3' cAMP
Pde4a
Cnp
Pde7b
Prkar2a
Prkar1a
PP1
CaMK2α
MEKK 1/2
MKK4
JNK
PKD
HDAC5
Eicosanoids
Ryr2
Casq2
Calr
Sarco/Endoplasmic reticulum
TNNI3
SERCA2a
PLN
AKAP
Glycogen
Gys1
Pygm
Glucose
Prkm
Glycolysis
Pyruvate
Fabp3
Fatty acid
CoA
Acsl1
Fatty-acyl-CoA
Carnitine
Acetyl-CoA
Mlycd
Acc1
Malonyl-CoA
AMPKα
PGC-1α
BCAA Catabolism
BCAAs
Bckdk
Bcat
BCKAs
Bckdh
Pdh
MICOS complex
Cpt1b
Cpt2
Mpc
Pc
Oxaloacetate
mt-FA-β oxidation
TCA cycle
Slc25a11
Slc25a42
Slc25a3
Sirt3
ETC complexes
Oxidative phosphorylation
PKCε
Camk2δ
ANT1
NCLX
Letm1
Slc family
Bnip3
Bax
Bcl2
DRP1
AKAP1
Taurine
Mfn1
SR/ER
IP3R
Vdac1
Mcu complex
Mrpl family of proteins
Ca²⁺
B
Inhibited
Activated
Log₂ fold change
-1.0 -0.8 -0.6 -0.4 -0.2 -0.1 0.1 0.2 0.4 0.6 0.8 1.0

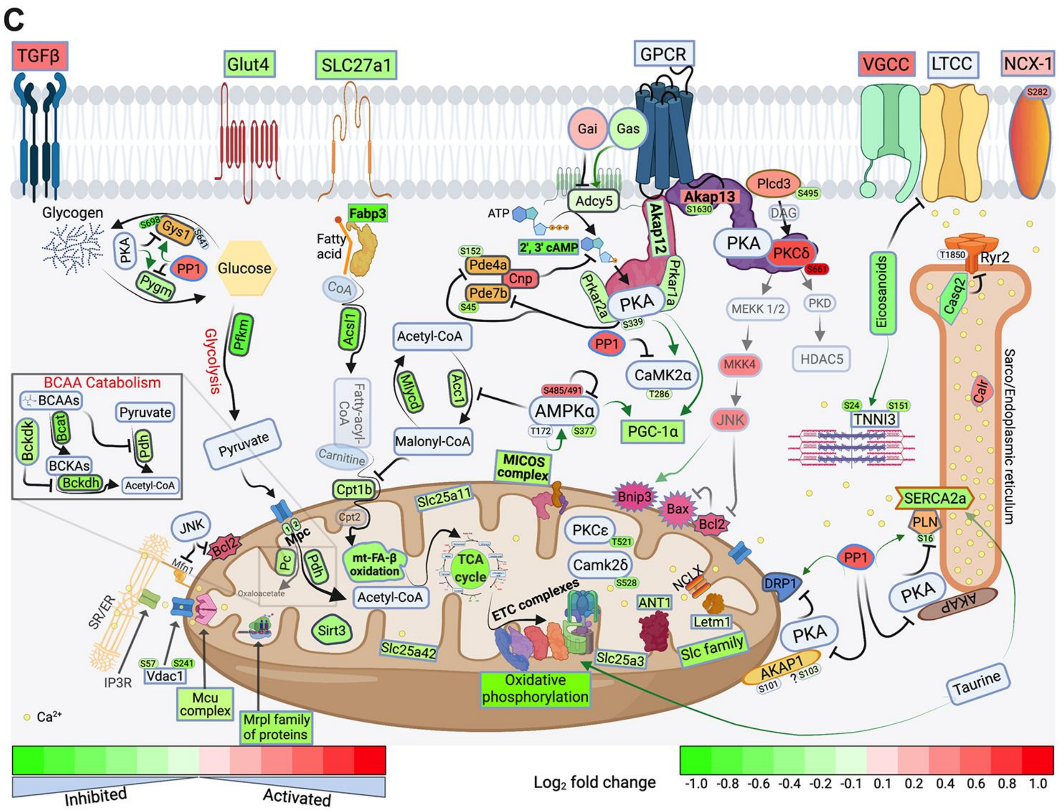

Figure S15. Schematic drawing highlighting metabolic pathways and calcium cycling as well as implicated signaling pathways in Sham (A), and those that changed in MOD vs Sham (B) and SHF vs Sham (C). Green arrows promote pathway or activity. Rectangles show signaling pathways or proteins that were inhibited or down-regulated (green), respectively, and those that were activated or up-regulated (red), respectively. The deeper the color, the higher is the intensity of activation/up-regulation or inhibition/down-regulation as shown by the scale bar at the bottom of the figure. Please refer to figure 8 for explanation.

Abbreviations: TGFβ: transforming growth factor beta, Glut4: glucose transporter family 4, Slc27a1: long-chain FA transport protein 1, Fabp3: FA binding protein 3, CoA: Coenzyme A, BCAA: branched-chain amino acids, BCKAs: branched-chain ketoacids, Vdac1: voltage dependent anion channel isoform 1, Mcu: mt-calcium uniporter, Cpt1b: carnitine-O-palmitoyltransferase isoform b, Sirt3: Sirtuin 3, Mrpl: mt-39S ribosomal proteins, Slc: mt-solute carrier family proteins, ANT1: ADP/ATP translocase 1, Letm1: proton/calcium exchanger, NCLX: sodium/calcium/lithium exchanger, PP1: protein phosphatase 1, DRP1: dynamin related protein 1, AKAP: A-kinase anchor protein, PLN: phospholamban, SERCA2a: Sarco/endoplasmic reticulum calcium ATPase, Calr: calreticulin, Casq2: Calsequestrin-2, Ryr2: ryanodine receptor isoform 2, NCX-1: sodium/calcium exchanger, LTCC: L-type calcium channel, VGCC: voltage gated calcium channels, GPCR: G protein-coupled receptor, Plcd3: 1-phosphatidylinositol 4,5-bisphosphate phosphodiesterase delta-3, Adcy5: adenylate cyclase type 5, Gai: G protein subunit alpha i2, Gas: GNAS complex locus, Pde4a: phosphodiesterase 4a, Pde7b: phosphodiesterase 7b, Gys1: glycogen synthase 1, Pygm: glycogen phosphorylase, muscle form, Pfkm: Phosphofructokinase, muscle type, Bcat: Branched-chain amino acid aminotransferase, Bckdh: 2-oxoisovalerate dehydrogenase, Bckdk: 3-methyl-2-oxobutanoate dehydrogenase [lipoamide] kinase, Pdh: Pyruvate dehydrogenase, Mfn1: mitofusin 1, IP3R: Inositolphosphate 3 receptor, Pc: Pyruvate carboxylase, Mpc: mitochondrial pyruvate carrier,

Slc25a3: mt-Phosphate carrier protein, Slc25a11: mt-2-oxoglutarate/malate carrier protein, Slc25a42: mt-coenzyme A transporter SLC25A42, PKCε: Protein kinase C, isoform epsilon, Camk2δ: Calcium/calmodulin kinase isoform 2 delta, Acsl1: Long-chain-fatty-acid--CoA ligase 1, Mlycd: Malonyl-CoA decarboxylase, Acc1: Acetyl-CoA carboxylase 1, Cnp: 2',3'-cyclic-nucleotide 3'-phosphodiesterase, and BNIP3: Bcl2 Nineteen Kilodalton interacting protein 3.

Reference

1. Yang, F.; Shen, Y.; Camp, D. G., 2nd; Smith, R. D., High-pH reversed-phase chromatography with fraction concatenation for 2D proteomic analysis. *Expert review of proteomics* **2012,** 9, (2), 129-34.
